# Commercially available antibiotic-laden PMMA-covered locking nails for the treatment of fracture-related infections - A retrospective case analysis of 10 cases

**DOI:** 10.7150/jbji.34072

**Published:** 2019-07-05

**Authors:** Alberto Jorge-Mora, Samer Amhaz-Escanlar, Sabela Fernandez-Pose, Adrián García-Iglesias, Fermín Mandia-Mancebo, Eloi Franco-Trepat, María Guillán-Fresco, Jesús Pino-Minguez

**Affiliations:** 1 Division of Traumatology, Santiago University Clinical Hospital, Santiago de Compostela, Spain; 2 Faculty of Medicine, University of Santiago de Compostela, Spain; 3 Musculoskeletal Pathology Group, Laboratory 18, Institute IDIS, Servicio Galego de Saúde, Santiago de Compostela, Spain

**Keywords:** Infection, long bone, antibiotic-laden PMMA-covered nail, fracture-related infection

## Abstract

**Introduction:** Fracture-related infections (FRIs) are a devastating complication. FRIs are challenging and should be addressed with a multidisciplinary approach. An FRI should be addressed surgically by non-viable bone debridement, local antibiotic deposition, minimization of dead space and fracture stabilization. Antibiotic-laden PMMA-covered nails are a viable option to face these complications. To demonstrate the safety and utility of commercially available antibiotic-laden PMMA-covered nails, we performed a review of the cases operated in our institution and a cost analysis to compare the cost of a commercial nail to other available alternatives.

**Material and methods:** We designed a retrospective study of consecutive cases to demonstrate the safety and efficacy of antibiotic-laden PMMA-covered commercial nails and designed a cost analysis of commercial coated nails compared to other custom-made alternatives.

**Results:** We treated seven tibias and three femurs. Nine patients fully fit the criteria for FRI. There was one case of reintervention because of persistent drainage. All fractures healed, and in the first year post-intervention, there were no signs or symptoms of infection. There were no complications related to the commercially available nail that was used. There is a small increase in the direct quantifiable cost in commercially available nails, but non-quantifiable cost should be assessed individually.

**Conclusions:** Commercially available antibiotic-laden PMMA-covered nails are a safe and useful treatment option for complicated cases of lower limb long bone reconstruction. The low complication rate and the straightforward technique compensate for the direct cost increase in most situations.

## Introduction

Long bone fractures are common 1, and it seems that there is an increase in the incidence of these fractures in elderly people 2. The infection rates of orthopaedic trauma procedures depend on the patient, the procedure and the injury sustained, and the infection rate differs among publications (2.9-14.2%) 3-5.

One of the major troubles regarding evidence in this field is the lack of consensus in older reports about the definition of fracture-related infection (FRI), which makes these publications difficult to integrate. The definition of FRI has not been validated in most of these studies 6. Recently, a consensus has arrived, with confirmatory and suggestive criteria to provide more homogeneity among publications 7. Similar to periprosthetic joint infection, these new criteria are based on clinical exams, medical history and surgical findings.

FRIs are challenging and should be addressed by a multidisciplinary approach: orthopaedic surgeons, plastic surgeons, infectious disease specialists, etc. All specialists should be involved in the management of these difficult-to-treat complications 8, 9. From an orthopaedic perspective, the presence of an FRI in a long bone should be managed surgically in different ways, but all strategies should include non-viable bone debridement, local antibiotic deposition, minimization of dead space and fracture stabilization 10. All of these strategies work to control and eradicate infection.

To achieve these goals, we can find new procedures and materials in the field of FRIs to facilitate treatment. The use of the reamer/irrigation/aspiration (RIA) system for intramedullary reaming (DePuy Synthes) has simplified the debridement procedure in long bones and has become an essential tool in our daily practice 11. The use of antibiotic-laden PMMA-covered nails has helped in the management of dead space, local antibiotic delivery and fracture stabilization 12-14.

The use of antibiotic-laden PMMA-covered nails has increased in the orthopaedic community since their popularization by Paley in 2002^15^, and now we can find them employed in numerous publications 12, 16-22. In most of these publications, as recently described by Wasko et al. 23, the nails used are custom-made with different techniques and procedures. We can find surface modification treatments (Gentamicin poly(D, L-lactide) matrix coating for tibia nails, silver coating or iodine coating) designed to minimize colonization in implants used in high-risk patients, but there are no treatments specifically for FRI 24, 25. Recently, some authors have advocated the use of PMMA custom-made plates for periarticular infected fractures 26, but their use is still limited.

Custom-made nails have the theoretical advantage of lower costs and the opportunity to select different antibiotics for local delivery, but not all antibiotics are able to be carried in PMMA. Antibiotics for this purpose should have specific physico-chemical properties 27: high solubility in water, thermostability to prevent degradation during polymerization, beneficial or non-deleterious to the mechanical properties of the PMMA, and should elute adequately from the polymeric matrix of the cement. There are also some desirable biological properties associated with the antibiotic selected^27^: high spectrum of action (gram-positives and gram-negatives), low-dose bactericidal action, low antibiotic resistance, low protein-binding rate, minor allergic characteristics and proper bone and cell penetration. All of these properties make gentamicin a good choice for obtaining an optimal concentration of antibiotic locally while causing minimal systemic complications 28.

**Figure 1:**
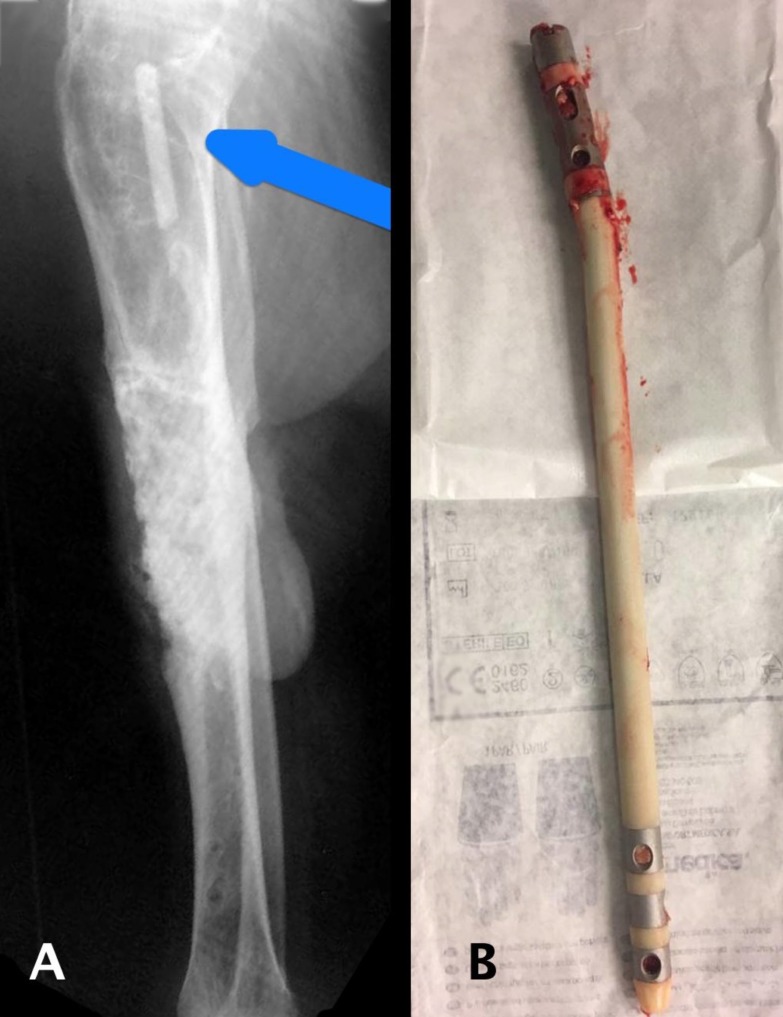
** A:** A lateral X-ray shows a broken piece of PMMA in the diaphysis of a tibia after a reconstructive process, indicated by the arrow. **B:** Image of a nail without mantle break after removal.

These custom-made nails have some disadvantages: first, their use is off-label, and custom-made implants should be explained to the patient with consent received prior to surgery. The surgeon should be familiar with the procedure to prevent errors that lead to complications (for example, selecting rifampicin or metronidazole, or mixing liquids with powder 29). It is important to note that all materials used at this point should be validated for medical use, and silicone moulds must be thermal resistant to minimize degradation during polymerization (plastics, polyvinylchloride). Second, the use of non-industrial devices, manually generated, is not reproducible in most cases, and the PMMA mantle and antibiotic elution can be different between implants constructed in the same manner 30. Third, some authors have created antibiotic-laden PMMA-covered locking custom-made nails 22, but not all described techniques provide a locking nail to achieve stability; some only act as spacers 31, 32. Finally, the mantle of PMMA could be removed easily during insertion of the custom-made implant, complicating future hardware extraction and generating free PMMA pieces/particles in the medullary canal (Figure 1 A) 31. Added to these inconveniences, we must include the waste of time dedicated to the fabrication of the nail, which increases surgical time and costs.

Recently, some manufactures have developed antibiotic-laden PMMA-covered locking nails as an alternative to custom-made nails. These implants have the great advantage of minimal time consumption. These nails are solid, not-cannulated, to prevent bacterial colonization and dispersion through the canal (this point should be considered because the absence of a rod to guide the nail may make its insertion difficult). The antibiotic-laden PMMA cover is uniform without weak zones (Figure 1 B). The nail is locked to increase stability. Finally, the elution of antibiotics from these nails is regular and is similar among implants. The two major disadvantages are limited antibiotic availability in the PMMA and the increased costs of the material.

To demonstrate the safety and utility of commercially available antibiotic-laden PMMA-covered nails, we have performed a review of the cases in our institution. We also performed a cost analysis to compare the cost of a commercial nail to a custom-made nail.

## Material and Methods

We designed a retrospective study of consecutive cases to demonstrate the safety and efficacy of an antibiotic-laden PMMA-covered commercial nail with a controlled elution of antibiotics. We also designed a cost analysis of commercial coated nails compared to a custom-made alternative in our institution.

This review was approved by our Ethics Committee.

### Patients

We completed a retrospective review of all patients who underwent an operation in our institution that included a commercially available antibiotic-laden PMMA-covered nail (Synicem clous, Synimed) with a minimum follow-up of 12 months.

We included 10 patients with a fracture sequela, including a subacute or chronic septic complication of a long bone in the lower limb. We included 9 males and 1 female. There were no patients excluded.

### Intervention

In all situations, we performed the same approach. Initially, all injuries were studied by clinical examination and complementary tests. Standard X-rays, blood tests and computed tomography with intravenous contrast images were routinely obtained. If necessary, we completed the medical records with MRI images.

**Figure 2:**
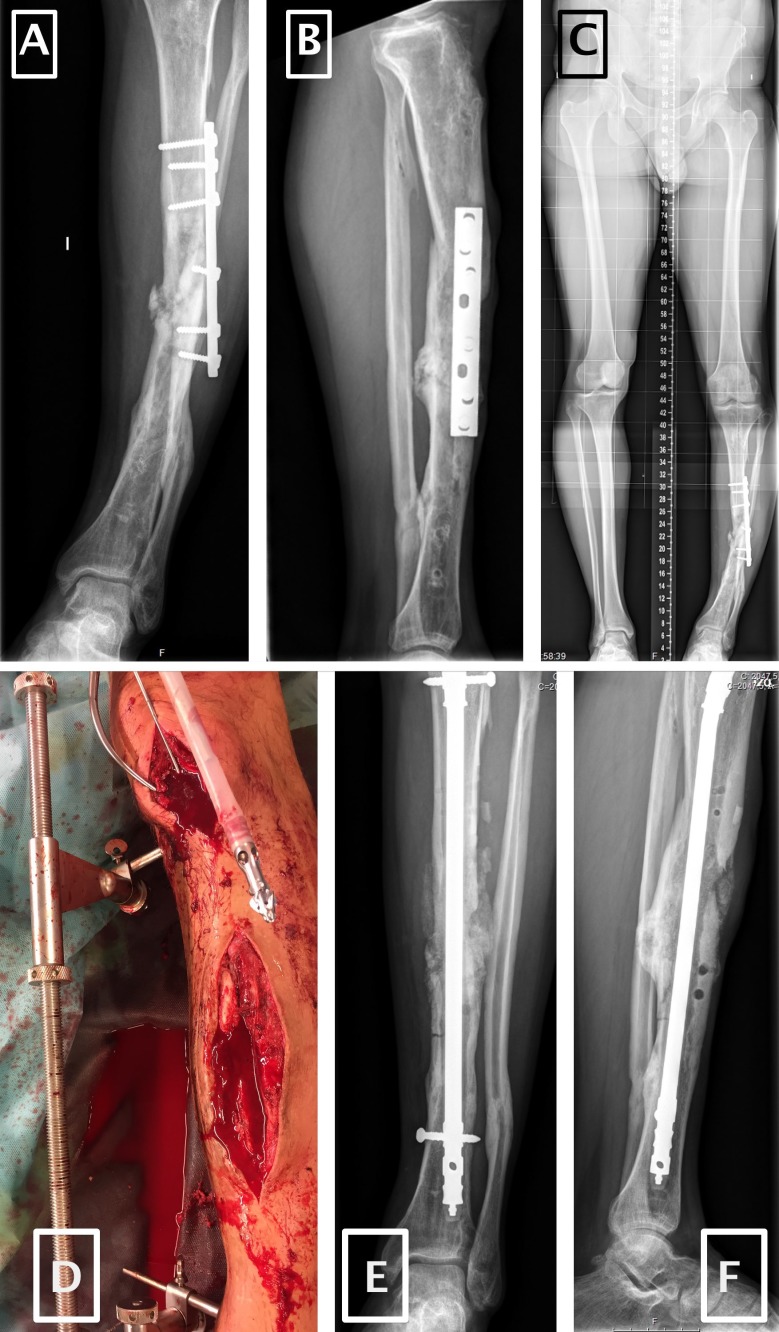
** Images that show the reconstructive steps of a tibial non-union.** Images **A** and **B:** Anteroposterior and lateral X-ray. Image **C:** Teleradiograph in full-weight. Image **D:** Intraoperative correction with a distractor and RIA reamer. Images E and F: postoperative anteroposterior and lateral X-ray after non-union treatment.

During the operation, we initially executed an aggressive resection of the dead and non-viable bone, and later we performed a complete reduction of the injury if needed, including temporal stabilization with the aid of an AO distractor. In the next step, we systematically use the RIA for intramedullary debridement 33. Afterwards, we implant the antibiotic-laden PMMA-covered nail (Synicem clous, Synimed) with locking screws. We select a nail 1 mm thinner than the diameter of the reamer selected for reaming. Depending on the case, injuries were managed in one or two stages (Figure 2). In cases in which we have to add PMMA for a bone defect, we load the cement with specific antibiotics according to the antibiogram and the pathogen characteristics.

A minimum of five tissue samples (bone and soft tissue) are obtained for culture.

If we decided to complete a two-stage procedure for bone defects (induced membrane technique 34) and we need to fill a bone gap with PMMA, there is a high risk of polymerization of the PMMA at the surface of the nail. To prevent this, we cover the exposed surface of the nail with a small quantity of bone wax. This will avoid a mantle break during PMMA removal in the second stage, and it will be easy to separate the nail from the PMMA.

In cases where soft tissue coverage is planned, plastic surgeons complete this step during the same surgery.

After the injury is healed, we remove the antibiotic-laden PMMA-covered nails according to the manufacturer's recommendations, unless the patient rejects hardware removal.

Antibiotic treatment is determined by operative cultures. Until we receive positive culture results, we systematically use vancomycin and meropenem for empirical treatment 35.

### Variables and outcomes

We include demographic characteristics for every patient. In all cases, we documented the bone affected, the bacteria responsible for the infection obtained by surgery cultures (minimum of two different samples) and the injury mechanism.

We diagnosed an FRI in those patients who fulfilled the criteria recently described by Metsemakers et al 7.

As a positive early outcome, we accepted a patient with a normalization of acute phase parameters (erythrocyte sedimentation rate, C-reactive protein and leukocyte levels), with no local pain, no clinical signs of infection and a consolidated fracture without the need for unplanned interventions (defined as an intervention not planned in the reconstructive process).

We also recorded complications related to the surgical process and complications related to the hardware (antibiotic-laden PMMA-covered nail).

### Cost analysis

We studied the cost difference between commercially available nails and custom-made nails. We studied the direct quantifiable costs in our environment. We included the cost of the nail, the PMMA used and the instruments needed to construct the PMMA nail, and the surgical time dedicated to the generation of the custom-made nail (estimated to be approximately 15 euros per minute by Antares Consulting y General Electric Healthcare in Spain).

## Results

### Patients

We included 10 consecutive patients with a median age of 45.3 years (range 22-63). We included 7 patients with a tibial injury and 3 patients with a femoral injury. Table 1 describes the cases and the bacteria responsible for the infection.

In our series, there were 6 patients with a septic non-union or a subacute infection after fracture fixation/stabilization and 3 patients with a posttraumatic chronic osteomyelitis with an acute exacerbation of the infection with a diaphyseal sequestrum that needed fracture stabilization after debridement.

All patients were type A and B of Cierny and Maden, and most injuries were stage IV (Table 1).

There was only one patient with negative cultures (Figure 2). This patient suffered a tibial non-union with a varus collapse. The patient was treated overseas multiple times. The infection was treated previously by an induced membrane technique 34 with no signs of consolidation and a varus deformity. In this case, because the preoperative test and operative strain were favourable, we chose a one-step procedure to treat the non-union.

Nine patients fulfilled the criteria for FRI, with a minimum of two operative cultures positive for the same pathogen.

### Complications

There were 2 complications recorded in our database.

One patient, because of medical treatment, suffered agranulocytosis. This condition was resolved by treatment adjustment and granulocyte colony stimulating factor.

There was one case of persistent drainage (after 3 days) that was treated by surgical lavage and direct closure with full resolution.

There were no complications related to the material, no PMMA mantle break, no complications during hardware removal, and in those cases where we used PMMA over the nail (as described in the surgical technique procedure), we had no polymerization between the PMMA and the surface. There were no unexpected events during PMMA removal (Figure 1B).

Two patients refused hardware removal with annual control in our outpatient clinic without complications at the last follow-up.

### Main outcomes

One year after the main procedure, all patients were alive.

Antibiotics were discontinued in all patients after a maximum of 3 months, with normalization of acute phase markers, no pain or new symptoms of infections, and no complementary tests suggesting complications.

All injuries healed without unplanned surgical procedures, except for one patient who needed an unexpected intervention because of persistent drainage, as previously described.

In those cases where nails were removed, cultures obtained during hardware removal were negative in all samples.

### Cost analysis

The direct cost of a custom-made nail, in those situations where there are no complications when performing the procedure, is 1747 euros (Table 2). The cost of a unitary commercially available antibiotic-laden PMMA-covered nail is 2820 euros. There is an increase in the cost by 1073 euros. This difference between the cost of both treatment options is the maximum obtained difference, excluding non-quantifiable costs, and can be minimized depending on the number of nails used. We also must deduct the wages of the orthopaedic team.

Non-quantifiable costs may minimize or change the previous cost difference. We have to include the costs derived from errors during the cementation of the nail, problems with the PMMA mantle, the surgical time dedicated to generating the nail (estimated as a minimum of 30 minutes), and an increase in complications associated with a longer operative time.

## Discussion

As previously discussed, FRIs are challenging for orthopaedic surgeons and also for a national health systems because of their burden, which doubles the standard cost of a primary fracture treatment and impairs the recovery of the patient^36^.

The recent development of a consensus in FRI 7, 37 has favoured consistency in the diagnosis of infection after fracture fixation, which is the first step toward improving homogeneity in publications and optimizing fracture management.

We have performed a retrospective study of our initial outcomes obtained with commercially available antibiotic-laden PMMA-covered nails to demonstrate their utility and safety. In our review, there were no hardware-related complications, even in those cases where patients refused removal. Surgical site infection in patients diagnosed with FRI was controlled in all situations. In those fractures where the antibiotic-laden PMMA-covered nail was used as a definitive fixation device, the fractures healed uneventfully. In those situations where the nail was used as a spacer, there were no hardware failures, and infection was controlled prior to the reconstructive surgery, as demonstrated by the negative intraoperative cultures and normalization of acute parameters.

There is no consensus about FRI cure, and there are no definitive parameters validated for this situation. Clinically, we can only discuss controlled infection, and most series use clinical evaluation, acute phase markers and complementary tests to evaluate a controlled infection 31. In our series, we chose these data to define a controlled infection. We also obtained fracture consolidation and good soft tissue coverage to improve function 38.

We cannot confirm that all of the improvement was secondary to the nail because management of FRI includes multiple steps that optimize the outcome. RIA reamer has proven to be an effective strategy for intramedullary infection control 33. The use of appropriate local and systemic antibiotics decreases bacterial ingrowth 39. A nail with controlled antibiotic elution (maintained for more than 3 weeks) allowed reproducibility between patients and straightforward treatment. The creation of multidisciplinary teams maximizes treatment outcomes 9, 40, 41. The use of antibiotic-coated nails is one more step, and commercially available nails seem to be safe and effective.

Pradhan et al.^20^, in a series of 21 patients treated because of an infection with an intramedullary antibiotic-laden PMMA-covered nail, reported 2 cases of hardware failure because of nail breakage, one case of non-union and several cases of lower limb discrepancy in patients treated with a custom-made nail without locking screws. We can see the importance of locking screws in periprosthetic fractures around the knee treated with a nail, and we can improve consolidation rates by increasing the number of locking screws 42, which may also be beneficial in infections. There are no reports of PMMA mantle complications in these series. In a recent review, there were multiple complications related to the hardware when managing infections with custom-made nails, and a series with a report of 20% PMMA mantle break. 31. To prevent these complications, an over-reaming of a minimum of 2 mm has been proposed 43, but this requires the use of small nails or aggressive reaming to use a wider nail. We did not have hardware complications in our 10-patient cohort. With the use of commercial nails, 1 mm of over-reaming is enough to prevent complications, maximizing the bone-implant contact and minimizing the dead space around the nail. Before the use of commercial nails, mantle break off was common in our practice, as can be seen in Figure 1A.

As we have shown, direct costs in antibiotic-laden PMMA-covered commercially available nails are higher than custom-made nails. The burden of an infection doubles the costs of a fracture treated without complications, with an average increased cost of 100,000 dollars in United States currency 36 and approximately 81,000 euros in European currency if we search for tibial infected fractures that need soft tissue coverage 44. In the case that we selected, a commercially available nail, we should only assume a maximum increase of 1% in costs in these situations.

At this time, we can only choose between two antibiotics in commercially available nails in our environment (gentamycin and tobramycin), but there are vancomycin nails available abroad. This point should be addressed by industry to provide more options to treat resistant bacteria because this is a handicap in our daily practice. In our series, we only chose gentamicin-coated nails. For cases infected by bacteria with specific resistance, for example, enterococcus with Gentamicin and Streptomycin High-Level Aminoglycoside Resistance (HLAR), we still use custom-made nails with adequate antibiotics. Despite this handicap, gentamycin-loaded PMMA is not inferior for infection control compared to vancomycin plus gentamycin-loaded PMMA 45.

There is recent evidence that supports the use of gentamicin covered nails to treat primary open tibial fractures 16, 25. Most of these studies use different surface treatments than our series (a fully resorbable antibiotic coating consisting of an amorphous poly(D,L-lactide)), and indications for these nails include prevention of infection in acute fractures or shaft fractures, but there is no indication for FRI. The use of antibiotic-laden PMMA-covered nails may also be an option for acute fractures with a high risk of infection. The use of these nails for all open fractures should be validated. In our study, we used an antibiotic-laden PMMA-covered nail for a patient who sustained an acute fracture in an osteomyelitic bone after debridement, and the outcome was favourable, as can be seen in image 3.

In our series, there were no cases of delayed healing when an antibiotic-laden PMMA-covered nail was used as definitive treatment, but the role of PMMA in fracture healing should be studied because PMMA can inhibit osteoblast activity 46.

There are multiple limitations in our study. First, it is a retrospective study of consecutive cases. The patients are heterogeneous but were treated with the same procedure. Our follow-up is short, and it was common that some of our patients continued with an infection that was activated in the future. There is no consensus about an FRI cure, and we have selected the most frequent parameters used in similar publications. Cost analyses are related to our country of practice and can vary substantially between countries and hospitals. Long-term studies with retained antibiotic-laden PMMA-covered nails should be made to confirm this short-term low complication rate in cases of refusal to remove hardware.

## Conclusions

Commercially available antibiotic-laden PMMA-covered nails are a safe and useful treatment option for complicated cases. The low complication rate and the straightforward technique compensate for the direct cost increase in most situations.

## Figures and Tables

**Figure 3:**
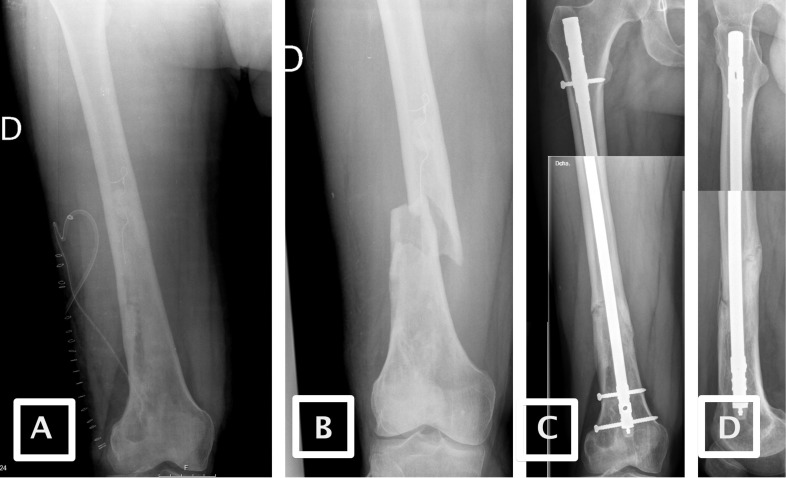
** Image A:** Postoperative image of the debridement of a sequestrum. Image **B:** Acute postoperative fracture after debridement. Images **C** and **D:** Anteroposterior and lateral X-ray after fixation.

**Table 1:**
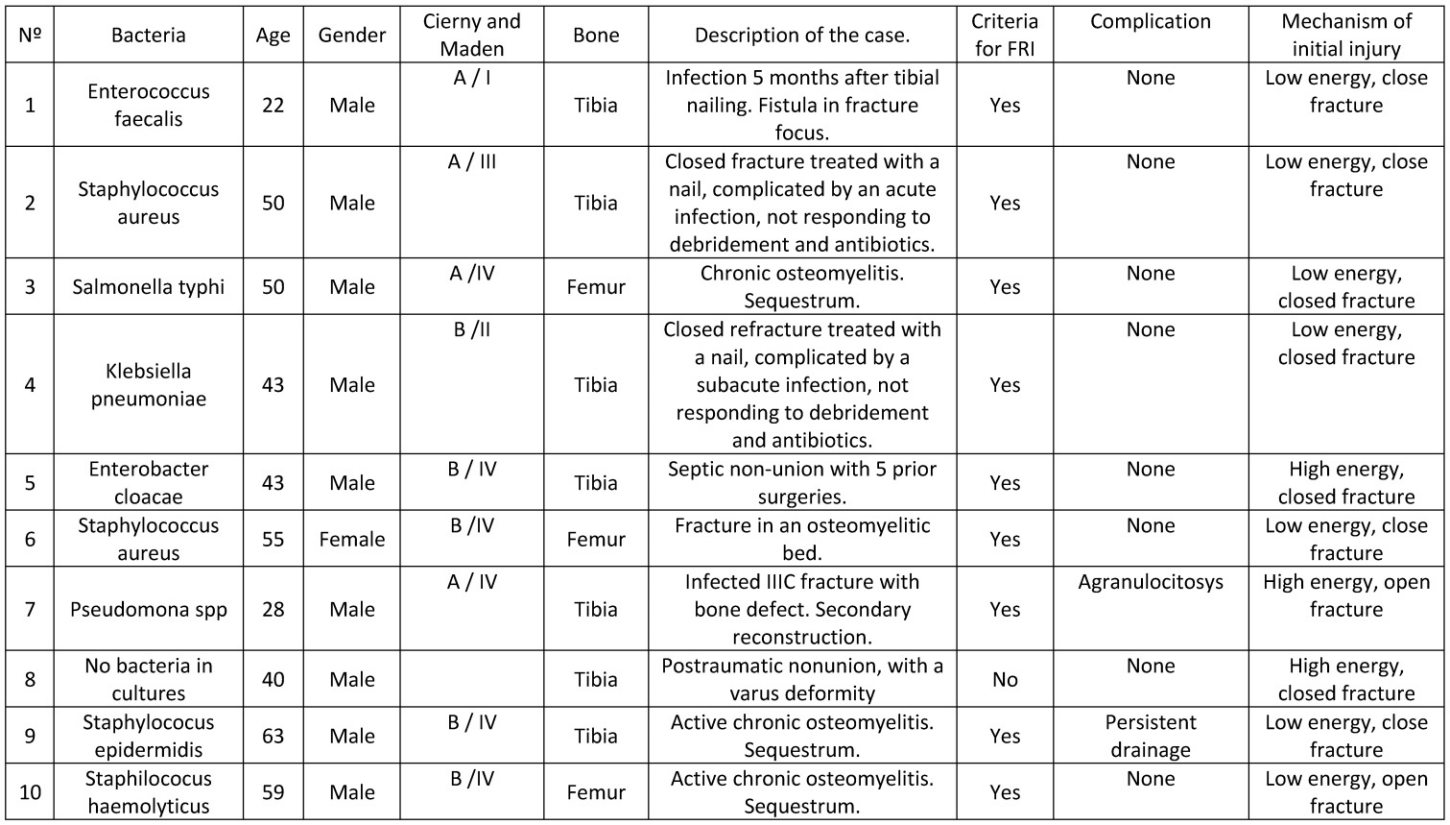
Demographic characteristics, bacteria responsible for infection and a description of the case.

**Table 2:**
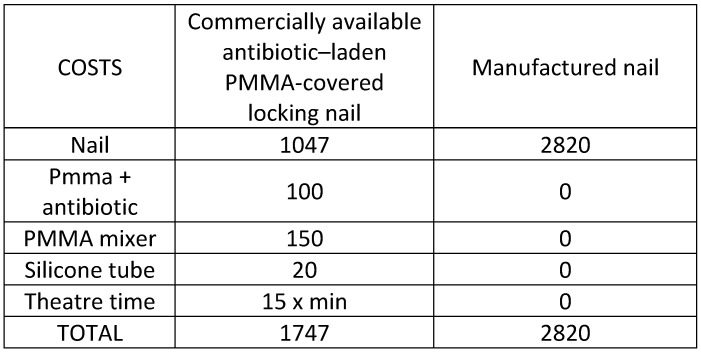
The cost of an antibiotic-laden PMMA-covered custom-made nail compared to a commercially available nail.
